# Antibiotic resistance genes profile of selected drinking water treatment plants in South Africa as impacted by different treatment stages

**DOI:** 10.1128/mra.00934-24

**Published:** 2024-12-19

**Authors:** Chimdi Mang Kalu, Khuthadzo L. Mudau, Lesoka Reneiloe Ntobeng, Vhahangwele Masindi, Memory Tekere

**Affiliations:** 1 Department of Environmental Science, College of Agriculture and Environmental Sciences, University of South Africa, Florida, South Africa; Rochester Institute of Technology, Rochester, New York, USA

**Keywords:** metagenomes, antibiotic resistance genes, drinking water, water treatment plants, water quality, South Africa

## Abstract

Water treatment plants treat raw water making it suitable for consumption. Treatment stages are linked to the quality status of the treatment. The classes of antibiotic resistance genes as impacted by treatment stages remain under explored. We report the classes of antibiotic resistance genes (ARGs) from shotgun metagenomes as influenced by treatment stages.

## ANNOUNCEMENT

The need for proper treatment of raw water to produce high-quality treated water that is void of antibiotic resistance genes (ARGs) cannot be overemphasized. Although the different stages of drinking water treatment plants (DWTPs) have been reported to play a satisfactory role in the provision of quality water, the persistent presence of ARGs is a global concern (1–4). The present study was undertaken to evaluate the impacts of different stages in five DWTPs in three provinces (Gauteng [A, D, and E], Limpopo [B], and Mpumalanga [C]) in South Africa on the abundance of ARGs.

Replicate samples were taken from the raw water, each stage of the treatments (Filtration—filtered water after coagulation, disinfection—after chlorine treatment, and final water—water obtained after the treatment) and sludge (waste obtained after treatment). Suitable volumes of the water samples (100 mL for raw water and 1 L for final water) were filtered through 0.22 µm polycarbonate membrane filters (PMF) immediately after collection. The PMF with collected microbial biomass was shredded using a sterile scalpel and used for DNA extraction. MN—NucleoSpin soil and sediment DNA extraction kit (Promolab Pty Ltd T/A Separations, RSA) were used following the manufacturer’s instructions. DNA was extracted in triplicates, and 5 µg genomic DNA used for further analysis was obtained by pooling the triplicates. The concentration/purity of DNA was determined using a NanoDrop spectrophotometer (Thermo Fisher Scientific, USA), and the quality was checked on a 1.5% agarose gel. The DNA sent to Agricultural Research Council (ARC) Biotechnology Platform for shotgun metagenomic sequencing had A260:A280 ratios between 1.8 and 2.0 and DNA concentrations of 20–150 ng/μL. Genomic Shotgun sequencing was performed on an MGI DNBSEQ-G400 sequencing instrument (Agricultural Research Council-Biotechnology Platform). The library preparation of the metagenomic shotgun data was done using MGIEasy Universal DNA Library Prep Set V1.0 (MGI Tech Co., China). The quality control of the library was done using a Qubit fluorometer (Life Technologies, Carlsbad, CA, USA) with the sequencing depth calculated to achieve 5 million reads. The DNA nanoball (DNB) was created by combining the pooled and circularized library, which was then loaded into a PE150 flow cell for sequencing in MGI DNBSEQ-G400 (MGI Tech Co., China) (2 × 150 bp). The raw Fastq files comprising the sequences from the MGI DNBSEQ-G400 sequencing platform were curated using the WGSA2.2 pipeline implemented in Nephele (v2.2.8) (5) using default parameters. A total of 751,581,663 quality reads (768,292–78,183,780), 65,672,955 contigs (48,659–8,484,722), and 16,791,477 N50 values (3,496–2,773,665) were obtained from all the samples. Metagenomes were screened for the presence of the genetic determinants of antibiotics in the WGSA2.2 pipeline implemented in Nephele (v2.2.8) using default parameters. Stacked bar charts were generated from the identified classes of ARGs using the Paleontological Statistics Software package Version 4 (PAST 4) (6).

**Fig 1:**
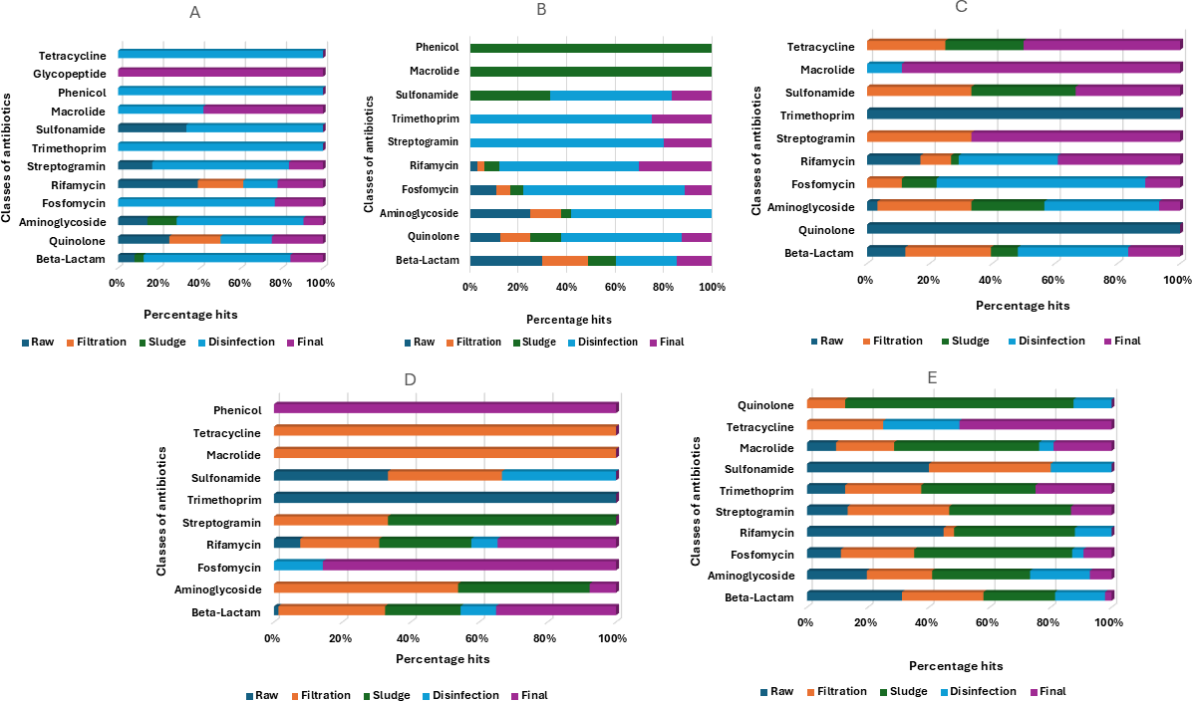
Classes of ARGs as impacted by the different treatment stages of five drinking water treatment plants.

Beta-lactam, rifamycin, aminoglycoside, streptogramin, sulfonamide, fosfomycin, macrolide, and trimethoprim were common in all the plants (7). Variations in the abundance of the ARGs belonging to these classes of antibiotics were observed across the plants and treatment stages. In comparison to the raw water, the treatment stages were pivotal in the abundance of ARGs. The disinfection stage and final stage of plants A, B, and C showed a more percentage hits of most of the classes of ARGs unlike the filtration stage of plants D and E (Fig. 1). The presence of ARGs in the final treated water raises great concern on the human health implications upon consumption of the water.

## Data Availability

In this study, the raw sequences were deposited in the National Center for Biotechnology Information Sequence Read Archive database as BioProject ID PRJNA1090776.
